# The Doctors That Meet in the Spring

**Published:** 1886-05

**Authors:** 


MIKADO MEDICUS; A "CONVENTION"—AL ADAPTATION.

(*By Dr. Merryman.*)

(Air: "The Flowers that Bloom in the Spring."

The doctors that meet in the spring, *tra la*,
To read papers on medical themes,
Find it a very difficult thing, *tra la*,
For each member an axe doth bring, *tra la*,
And to grind it, his prime object seems.
Hence most of the session is spent in discussion
Of irrelevant matters and things.
And that's what I mean when I say or sing,
"God bless the doctors that meet in the spring,"
*Tra la*, *tra la*, *tra la*, *tra la*,
Hurrah for the doctors that meet in the spring!

